# Communication of patients’ and family members’ ethical concerns to their healthcare providers

**DOI:** 10.1186/s12910-023-00932-x

**Published:** 2023-07-29

**Authors:** Mariam Noorulhuda, Christine Grady, Paul Wakim, Talia Bernhard, Hae Lin Cho, Marion Danis

**Affiliations:** 1 National Institutes of Health Clinical Center, 10 Center Drive, Bethesda, MD 20892 USA; 2 Thomas Jefferson University, 26 E Red Bank Ave, Woodbury, NJ 08096 USA; 3 Harvard Medical School, 25 Shattuck St, Boston, MA 02115 USA

**Keywords:** Clinical ethics, Communication, Professional-patient relations, Family members, Surveys and questionnaires

## Abstract

**Background:**

Little is known about communication between patients, families, and healthcare providers regarding ethical concerns that patients and families experience in the course of illness and medical care. To address this gap in the literature, we surveyed patients and family members to learn about their ethical concerns and the extent to which they discussed them with their healthcare providers.

**Methods:**

We surveyed adult, English-speaking patients and family members receiving inpatient care in five hospitals in the Washington DC-Baltimore metropolitan area from July 2017 to March 2020. Descriptive statistics were used to determine the frequency, comfortableness, and helpfulness of discussions regarding ethical concerns experienced when sick or receiving medical care. Univariable and multivariable stepwise logistic regression models were used to identify associations between healthcare provider and respondent characteristics and attitudes and (1) the likelihood of speaking to a healthcare provider about their ethical concern and (2) their level of comfort during these discussions.

**Results:**

Of 468 respondents who experienced ethical issues, 299 (64%) reported discussing the situation with a member of their healthcare team; 74% (197/265) of respondents who had such a discussion found the discussion comfortable, and 77% (176/230) of respondents found the discussion helpful. To make discussions more comfortable and helpful, respondents proposed suggestions in open-ended responses involving (1) content and quality of communication; (2) positive healthcare provider qualities such as empathy, open-mindedness, knowledge, honesty, and trustworthiness; and (3) other contextual factors including having adequate time and available resources.

**Conclusions:**

Patients and families often have ethical concerns that they discuss with clinicians, and they want clinicians to be routinely receptive and attentive to such discussions.

**Supplementary Information:**

The online version contains supplementary material available at 10.1186/s12910-023-00932-x.

## Background

Patients, family members, and healthcare providers regularly face ethically challenging situations, situations in which they feel uncertain about the right choice to make, during illness and medical care. Several studies have explored ethical concerns from the perspectives of physicians, nurses, social workers, and other healthcare providers [1–3]. However, to our knowledge, few studies have surveyed patients and their families about their experiences with ethically challenging situations while living with illness and receiving medical care [4].

Moreover, little is known regarding the tendency of patients and families to communicate with their healthcare providers about the ethical concerns they might have during such challenging circumstances. In ethically complex situations, good communication can help manage emotions, promote understanding of medical information, and allow for better identification of patients’ needs, perceptions, expectations, and values [5–7]. Good communication has also been shown to improve patient and family satisfaction, adjustment to illness, adherence to medical treatment, and overall clinical outcomes [8, 9].

The lack of data about communication regarding ethical concerns makes it difficult for healthcare providers to assess and improve their efforts to sufficiently address these concerns. To address this gap in the literature, as part of a multi-hospital survey of patients’ and family members’ experiences of ethical concerns [10], we aimed to describe whether and to whom patients and families spoke when faced with an ethical concern and how comfortable and helpful they found these discussions.

## Methods

We conducted a cross-sectional, mixed-methods survey examining patients’ and family members’ experiences with ethical issues [10]. The study as a whole examines (1) the frequency and type of ethical concerns experienced by patients and families, (2) the extent to which patients and families spoke with their healthcare providers about these concerns, (3) how comfortable and helpful these discussions were, and (4) to what extent patients and families were aware of and interested in speaking with clinical ethics consultants. We present the results for (2) and (3) in this paper.

### Participants

We surveyed participants from July 2017 to March 2020 at five hospitals in the D.C.-Baltimore metropolitan area: a public university hospital, a private university hospital, a US military hospital, a private hospital, and a religiously affiliated hospital. Participants were adult, English-speaking hospitalized patients and family members. Recruitment took place in general medicine and surgical wards, and Intensive Care Unit (ICU) waiting rooms.

Patients were given the first opportunity to participate in medical and surgical wards; family members were approached directly without approaching the patient in the ICU except in rare circumstances when an ICU patient could respond. To make participation minimally burdensome, patients were given the option of completing the survey with the help of a family member or a research assistant. Patients who lacked cognitive capacity, were sleeping, eating, receiving medical care, in isolation for infection control, or who appeared to be in pain or distress were not invited to participate. Family members were surveyed if a patient wished to have a family member take the survey instead or if a patient was critically ill. Our survey response rate was 37% overall (range, 28–72% among the five hospitals). Additional details about survey administration are reported elsewhere [10].

### Survey instrument and measures

Participants were asked whether they had encountered any of the following situations in their own or their family member’s illness or medical care that might raise ethical uncertainty: limiting life-sustaining treatment, advance care planning, changing goals to comfort care, uncertainty about a family member’s decision making capacity, privacy from the family, family disagreements, healthcare access and cost, disagreements with doctor recommendations, clinical trial enrollment, genetic testing, dangerous behavior in a family member, the medical team withholding information, and reproductive decisions. Participants were then asked to think about the ethically challenging situation they remembered best and respond to a subset of survey questions that were designed to inquire about whether the respondent had discussed the situation they recalled best with a healthcare provider and/or other individuals, the types of providers they spoke to, who had initiated these discussions, their comfort level at the start of the discussions, the overall helpfulness of the discussions, as well as respondent and healthcare provider characteristics and attitudinal factors that may have motivated the respondent to speak to a healthcare provider or influenced their level of comfort. Factors that we predicted to possibly make individuals more inclined to speak with a healthcare provider and/or feel comfortable were labeled as positive and factors that we predicted to possibly make respondents less inclined to speak to a healthcare provider and/or feel comfortable were labeled as negative (see attitudinal factors listed in Table 2). We also collected sociodemographic variables, such as age, birthplace, insurance status, and race/ethnicity. The survey instrument is published elsewhere as an appendix [10]. 

### Data analysis

Descriptive statistics describe the percentage of patients and families who reported speaking with healthcare providers, which party initiated the discussion, the respondent’s comfort level at the start of the discussion, and the overall helpfulness of the discussion. We modeled associations between respondent sociodemographic variables and their ratings of respondent- and healthcare provider-related attitudinal factors listed in Table 2. Finally, we modeled associations between 15 healthcare provider/respondent-related independent variables and the following dependent variables: (1) whether the survey respondent spoke to a healthcare provider about their ethical concern and (2) the survey respondent’s level of comfort during these discussions. The 15 independent explanatory variables consisted of: age, gender, marital status, birthplace, race/ethnicity, religious preference, employment, education level, household income, health insurance (type and amount), number of positive and negative respondent-related attitudinal factors, and number of positive and negative healthcare provider-related attitudinal factors.

To model the final associations, we constructed a summary score for positive and negative respondent- and healthcare provider-related attitudinal factors (listed in Table 2). The association between whether the respondent spoke to a healthcare provider (dichotomized as yes/no) and 15 healthcare provider and respondent characteristics was examined in two ways: (1) through univariable analysis; and (2) through a multivariable stepwise logistic regression approach with the comparison of corresponding area under the receiver operating characteristic curves (AUROCs) to determine the “best” set of predictors of speaking to a healthcare provider. Univariable analyses determined which specific attitudinal factors were predictors of speaking to a healthcare provider. The same method was used for the association between the level of comfort during the discussion of the ethical concern (dichotomized as comfortable vs. uncomfortable) and the 15 explanatory variables.

For the open-ended responses regarding the comfort level and helpfulness of the discussion, codes were developed using abductive analysis, a type of qualitative content analysis that combines elements of induction and deduction [11, 12]. One researcher (MN) and one research assistant (TB) created preliminary codes based on responses from the first batch of surveys. MN coded all participant responses and revised the preliminary codebook with the assistance of TB and another researcher (MD). Using the revised codebook, MN and TB independently coded all the data and resolved disagreements by discussion. Any remaining disagreements were resolved by discussion among the three coders (MN, TB, and MD) until consensus was reached. Responses could receive multiple codes, and codes were not mutually exclusive. The coding schemes for the open-ended responses are reported in Additional file 1. Any edits for readability are minor.

### Human subjects protection

This study was determined exempt by NIH Office of Human Subjects Research Protection, as well as by the Institutional Review Boards of each of the participating hospitals. No personally identifying information was collected in this survey.

## Results

Of 671 participants, 485 were patients, 182 family members, and 4 did not specify. Patients and family members were demographically diverse with respect to race, gender, birthplace, income, and religious preference (Table 1). The vast majority were born in the US and had health insurance.

**Table 1:** Respondent (patient or family member) demographics^a^

	Patients (*N*=485)	Family members (*N*=182)
Age		
18-44	85 (18%)^b^	48 (26%)
45-64	179 (37%)	71 (39%)
65+	171 (35%)	38 (21%)
Gender		
Male	238 (49%)	55 (30%)
Female	225 (46%)	115 (63%)
Marital status		
Married/living with partner	201 (41%)	111 (61%)
Widowed	50 (10%)	10 (5%)
Divorced/separated	66 (14%)	19 (10%)
Single	139 (29%)	29 (16%)
Birthplace		
U.S.	396 (82%)	153 (84%)
Outside the U.S.	63 (13%)	17 (9%)
Race		
White	180 (37%)	87 (48%)
Black/African American	222 (46%)	54 (30%)
Hispanic	23 (5%)	11 (6%)
Asian	11 (2%)	8 (4%)
Multiple/other	23 (5%)	11 (6%)
Education		
High school or less	137 (28%)	35 (19%)
College	212 (44%)	94 (52%)
Graduate school	109 (22%)	39 (21%)
Household income		
<$25,000	105 (22%)	21 (12%)
$25,000-$49,999	83 (17%)	21 (12%)
$50,000-$99,999	102 (21%)	54 (30%)
>$100,000	93 (19%)	52 (29%)
Religious preference		
Protestant/other Christian	237 (49%)	82 (45%)
Catholic	94 (19%)	44 (24%)
No religious preference	87 (18%)	28 (15%)
Other	38 (8%)	17 (9%)
Employed		
Yes	177 (36%)	115 (63%)
No	283 (58%)	54 (30%)
Source of insurance		
Employer	111 (23%)	74 (41%)
Plan paid for by self or family	29 (6%)	26 (14%)
Medicare	115 (24%)	31 (17%)
Medicaid	72 (15%)	11 (6%)
Military or veteran	29 (6%)	7 (4%)
Multiple/other	81 (17%)	15 (8%)
None	13 (3%)	3 (2%)

^a^Table 1 has also been published in *AJOB Empirical Bioethics* as part of a report describing the major findings of our survey

^b^Four respondents did not indicate whether they are a patient or a family member. Percentages do not add up to 100 because missing data were not included in the table

Seventy percent (468/671) of the patients and family members from our survey described an ethically challenging situation that they had experienced in their illness or healthcare encounters. When asked to think about the ethically challenging situation that they remembered best, 56% of respondents either agreed or strongly agreed that they wanted the advice of a healthcare provider, while 33% wanted the advice of someone other than their healthcare provider (Table 2). 69% of respondents either agreed or strongly agreed that the healthcare provider who was involved was kind, and 65% agreed or strongly agreed that the healthcare provider was trustworthy (Table 2). Respondents who were unsure whether the situation was important were more likely to be Black or African American than white (34% versus 11%, *p* = 0.0001; see Additional file 2).

**Table 2:** Attitudinal factors (Question 5 of the survey asked: “In thinking about the [ethically challenging situation you remember best], please tell us how strongly you agree or disagree with the following statements.)

**A. Respondent-related attitudinal factors (*N*=468)**^**a**^							
Factor (+/-)^b^	Strongly agree	Slightly agree	Slightly disagree	Strongly disagree	Missing	*p*-value for speaking with healthcare provider	*p*-value for comfortable discussion
I wanted advice from the healthcare provider (+)	189 (40%)	76 (16%)	36 (8%)	53 (11%)	114 (24%)	0.013	0.16
I wanted to talk with someone else instead of the healthcare provider (-)	94 (20%)	60 (13%)	67 (14%)	137 (29%)	110 (24%)	0.59	0.0001
I didn’t feel ready to have this discussion (-)	73 (16%)	69 (15%)	59 (13%)	159 (34%)	108 (23%)	0.059	0.0001
I thought it was better for someone else in my family to talk with the healthcare provider (-)	72 (15%)	63 (13%)	55 (12%)	170 (36%)	108 (23%)	1.0000	0.0003
I didn’t know which person to talk to (-)	61 (13%)	83 (18%)	56 (12%)	158 (34%)	110 (24%)	0.50	0.0001
I felt powerless to voice my opinion (-)	59 (13%)	36 (8%)	61 (13%)	210 (45%)	102 (22%)	0.055	0.0001
I wasn’t sure if this situation was important (-)	39 (8%)	46 (10%)	55 (12%)	215 (46%)	113 (24%)	1.0000	0.39
I felt like I didn’t have enough privacy (-)	30 (6%)	36 (8%)	61 (13%)	230 (49%)	111 (24%)	0.86	0.26
I was embarrassed (-)	29 (6%)	29 (6%)	47 (10%)	254 (54%)	109 (23%)	0.28	0.0006
**B. Healthcare provider-related attitudinal factors (*N*=468)**^**a**^							
Factor (+/-)^b^	Strongly agree	Slightly agree	Slightly disagree	Strongly disagree	Missing	*p*-value for speaking with healthcare provider	*p*-value for comfortable discussion
Healthcare provider seemed trustworthy (+)	233 (50%)	72 (15%)	30 (6%)	26 (6%)	107 (23%)	0.35	0.0008
Healthcare provider seemed kind (+)	225 (48%)	96 (21%)	18 (4%)	20 (4%)	109 (23%)	0.12	0.0001
Healthcare provider spoke in a way that was easy for me to understand (+)	224 (48%)	78 (17%)	26 (6%)	30 (6%)	110 (24%)	1.0000	0.0001
Healthcare provider was a good listener (+)	190 (41%)	98 (21%)	35 (7%)	38 (8%)	107 (23%)	0.62	0.0001
Healthcare provider seemed to fully understand the situation (+)	182 (39%)	102 (22%)	37 (8%)	36 (8%)	111 (24%)	0.87	0.0001
Healthcare provider seemed very busy (-)	106 (23%)	113 (24%)	54 (12%)	84 (18%)	111 (24%)	0.092	0.38
Healthcare provider was part of the problem (-)	58 (12%)	56 (12%)	47 (10%)	197 (42%)	110 (24%)	0.38	0.0001

^a^The number of individuals who experienced at least one ethical concern

^b^Factors that were expected to make individuals more inclined to speak with a healthcare provider and/or feel comfortable are labeled as positive and factors that were expected to make respondents less inclined to speak to a healthcare provider and/or feel comfortable are labeled as negative

### Discussions of ethical concerns with healthcare providers

Of those who reported experiencing an ethical concern *and* who responded regarding who, if anyone, they spoke with, the majority (74% or 299/404) spoke to a healthcare provider about the ethical concern they remembered best (Table 3). The types of healthcare providers spoken to, in descending order, were the respondent’s or their family’s main doctor in the hospital, their regular doctor, a nurse, and a social worker (Table 4). 65% (193/299) of respondents who spoke to a healthcare provider about their ethical concern also consulted a family member or friend.

**Table 3:** Who did respondent speak with?

	Respondents who experienced ethically challenging situations (*N*=468)
Only with healthcare provider^a^	106 (23%)
Only with non-healthcare provider individuals	65 (14%)
Healthcare provider and non-healthcare provider individuals	193 (41%)
No one	40 (9%)
Missing	64 (14%)

^a^Healthcare providers include the respondent’s or family member’s regular doctor, their main doctor in the hospital, a nurse, a social worker, or any other healthcare providers not listed. Other non-healthcare provider individuals include family members or friends, clergy member or religious advisors, or any other non-healthcare provider individuals

**Table 4:** What types of individual(s) did the respondent speak with?

	Respondents who reported speaking to someone (N=364)
A healthcare provider	299 (82%)^a^
Your main doctor in the hospital	149 (41%)
You or your family’s regular doctor	125 (34%)
A nurse	95 (26%)
A social worker	73 (21%)
Another healthcare provider not listed above	64 (18%)
I don’t know	19 (5%)
A family member or friend	239 (66%)
A clergy member or religious advisor	48 (13%)
Other	28 (8%)

^a^Percentages add up to more than 100 since many respondents spoke to a wide array of individuals

In the univariable analysis involving attitudinal factors, the only association was a weak association (*p* = 0.013) between those who wanted advice from the provider and speaking to a healthcare provider (Table 2). No other factor was associated with speaking to a healthcare provider (Table 2). In the multivariable logistic regression with the 15 candidate explanatory variables, speaking to a provider was not associated with any patient or family sociodemographic variables (Additional file 3). However, respondents who agreed with a greater number of positive statements relating to themselves (see Table 2) were more likely to speak to a healthcare provider about their ethical concern (*p*-value = 0.0009, AUC = 0.62, AUC 95% CI: 0.55, 0.69) (Additional file 3).

Of the 299 patient and family members who reported discussing the ethical concern with at least one healthcare provider, data on who started the discussion were missing for 30 respondents. Of the remaining 269 respondents, 42% reported that they themselves initiated the discussion; 31% that the healthcare provider initiated the discussion; 10% that both they and the healthcare provider together initiated the discussion; and 17% that the discussion was initiated by someone else. Respondent sociodemographic variables were not associated with whether the respondent or provider initiated the discussion.

### Comfort level of discussions

Most (89% or 265) of the 299 patient and family members who discussed their concern with a healthcare provider reported how comfortable they were with the conversation. Of these 265 respondents, 35% felt very comfortable, 39% comfortable, 18% uncomfortable, and 8% very uncomfortable during the discussion.

In the univariable analyses, respondents who felt they could voice their opinion; knew who to talk to; wanted to talk with the healthcare provider; and felt ready to have the discussion were more likely to feel comfortable discussing their ethical concern with the provider (all *p*-values < 0.0001) (Table 2). Respondents who disagreed that it was better for someone else in their family to talk with the healthcare provider (*p* = 0.0003) and disagreed with feeling embarrassed (*p* = 0.0006) were also more likely to report feeling comfortable with the discussion (Table 2). Regarding healthcare provider attitudinal factors, respondents who felt the healthcare provider understood the situation; was not part of the problem; was a good listener; spoke in a way that was easy to understand; was kind (all *p*-values < 0.0001); and was trustworthy (*p* = 0.0008) were more likely to feel comfortable discussing their ethical issue with them (Table 2). Respondent sociodemographic variables were not associated with the respondent’s comfort level speaking to a healthcare provider (Additional file 4), nor were the factors of wanting advice from the healthcare provider (*p* = 0.16); feeling like they didn’t have enough privacy (*p* = 0.26); being unsure if the situation was important (*p* = 0.39); or the healthcare provider seeming very busy (*p* = 0.38) (Table 2).

In the multivariable logistic regression, respondents who agreed with more positive statements regarding themselves, and who disagreed with more negative statements regarding themselves (see Table 2), were more likely to feel comfortable discussing their ethical concern with the provider (*p*-value < 0.0001) (Additional file 4). Respondents who agreed with more negative statements about the healthcare provider, and who disagreed with more positive statements about the healthcare provider (see Table 2), were less likely to feel comfortable (*p*-value = 0.0001) (overall AUC = 0.82, AUC 95% CI: 0.76, 0.88) (Additional file 4). Although the evidence is fairly weak after accounting for multiple statistical tests, respondents who had less than a high school education were less likely to feel comfortable discussing their ethical concern with the provider than respondents from other educational levels (*p* = 0.03) (Additional file 4).

In open-ended replies, 113 survey respondents offered suggestions about what would have made their conversation about their ethical concern more comfortable (Table 5). Respondents most frequently suggested that the discussion would have been more comfortable if the healthcare provider had given more or different information, had been more empathetic, and had more time for discussing or thinking about their ethical issue.

**Table 5:** Suggestions for making discussions with healthcare providers more comfortable

Suggestions	Responses (*N*=113)
**Contextual factors**	66 (58%)^a^
- Having adequate time/timing	
- Providing sufficient resources or referrals	
- Being prepared or ready to have the discussion	
- Having a previously established relationship with the healthcare provider	
- Having privacy	
- Having family present or involved	
**Healthcare provider qualities**	49 (43%)
- More empathetic	
- More open-minded	
- More knowledgeable and experienced	
- More honest and transparent	
- More trustworthy	
**Content and/or quality of communication**	46 (41%)
- Providing more or different information	
- More listening	
- Better or clearer communication	
- More proactive communication	

^a^Percentages add up to more than 100 since many respondents noted more than one type of factor that would have made the discussion with the healthcare provider more comfortable

## Helpfulness of discussions

The majority (77%) of the 230 respondents who reported whether the discussion about their ethically challenging situation was helpful, reported finding the discussion helpful, while 23% reported not finding the discussion helpful. Sixty-nine responses were missing or uninterpretable.

In open-ended comments (*N* = 189) describing why discussions with the healthcare provider were helpful (Table 6, listed in order of frequency), the overwhelming majority (76%) referred to receiving valuable, helpful information that answered their questions, explained their options, and/or gave them a better understanding of the situation. The next most repeatedly cited factors that made discussions helpful were the healthcare provider’s knowledge and/or experience and empathy; and the discussion’s utility in helping their decision-making about their ethical issue.

**Table 6:** Factors that made the discussion with the healthcare provider helpful

Factors	Responses (*N*=189)
**Content and/or quality of communication**	144 (76%)^a^
- Provided helpful information	
- Communicated well or clearly	
- Listened	
**Healthcare provider qualities**	72 (38%)
- Knowledgeable or experienced	
- Empathetic	
- Honest or transparent	
- Patient	
- Trustworthy	
- Put respondent at ease	
- Open-minded	
**Discussion involved helpful action**	48 (25%)
- Assisted with decision-making	
- Facilitated access to helpful resources	
- Intervened effectively	
**Time and availability of the healthcare provider**	3 (2%)

^a^Percentages add up to more than 100 since many respondents noted more than one type of factor that made the discussion with the healthcare provider helpful

Sixty-five respondents described why discussions with the healthcare provider were not helpful; 52% referred to the content and/or quality of communication, stating that the provider did not provide helpful information, did not listen, communicated unclearly, or did not actually communicate with them; 52% found the qualities and character of the healthcare provider a barrier because the provider was single-minded or dismissive, unempathetic, not knowledgeable or experienced, untrustworthy, or hurrying. Finally, 23% found the discussion unhelpful because no solution was reached because of other contextual factors such as the lack of resources, and lack of privacy.

Suggestions for making discussions more helpful are shown in Table 7.

**Table 7:** Suggestions for making discussions with healthcare providers more helpful

Suggestions	Responses (*N*=105)
**Content and/or quality of communication**	50 (48%)^a^
- Providing more or different information	
- Better or clearer communication	
- More listening	
**Healthcare provider qualities**	43 (41%)
- More empathetic	
- More knowledgeable and experienced	
- More open-minded	
- More honest and transparent	
- More patient	
**Contextual factors**	38 (36%)
- Having adequate time/timing	
- Providing sufficient resources or referrals	
- Being prepared or ready to have the discussion	
- Having a previously established relationship with the healthcare provider	
- Having privacy	
- Having family present or involved	

^a^Percentages add up to more than 100 since many respondents noted more than one type of factor that would have made the discussion with the healthcare provider more helpful

## Discussion

This study examines patients’ and family members’ reports of communication with healthcare providers regarding ethical concerns that arise during their own or their family members’ illness and healthcare. The majority of survey respondents reported an ethical concern and most reported speaking to a healthcare provider, often in addition to speaking with family and friends.

Our findings demonstrate that patients and family members find having discussions with a wide variety of individuals, especially healthcare providers, helpful when faced with ethical questions and concerns during illness and medical care. However, a substantial minority, about one out of four individuals, did not speak to a healthcare provider, and approximately another quarter did not find the discussion with healthcare providers to be helpful. Notably, sociodemographic variables, like race and ethnicity, were not associated with the likelihood that the respondent spoke to a healthcare provider.

In thinking about the ethically challenging situation they remembered best, the majority of survey respondents were not embarrassed, felt like they had enough privacy, understood the situation was important, did not feel powerless to voice their opinion, and wanted advice from the healthcare provider. The only association found between the individual attitudinal factors and respondent sociodemographic variables was respondent race which was significantly associated with whether they were unsure if the situation was important. In a setting where they are often underrepresented, minority patients and families may feel less assertive and more passive in their healthcare encounters [13, 14]. Power imbalances may make minority patients feel intimidated or unqualified and lead them to doubt the importance of their ethical concerns. The finding lends support to the recommendation that healthcare organizations should create an environment where patients and families from all backgrounds feel comfortable and confident speaking up about their concerns and moral distress. One way to promote comfort for minority patients and family members is to diversify the healthcare workforce [15]. Aside from more listening, providers should also be more attuned to actively eliciting patient and family concerns and helping them understand that their concerns are legitimate and appropriate topics for discussion.

The majority of respondents said their healthcare provider seemed trustworthy and kind, spoke in a way that was easy to understand, was a good listener, seemed to fully understand the situation, and was not a part of the problem. Respondents varied more widely with whether they felt the healthcare provider seemed very busy. Although this variable was not associated with the likelihood that respondents spoke up, busy clinician schedules could, and likely do, affect communication since trust-building and in-depth conversations, particularly those required for ethically challenging situations, require time. Many respondents, in fact, thought more time with the healthcare provider would have made discussing their concern more comfortable. These results build on existing evidence on the importance of unrushed visits and more time in promoting patient satisfaction and trust [16, 17].

Patients and families reported that they were more likely than healthcare providers to initiate this discussion. These findings are consistent with the cultural shift in medical practice in which patients play an increasingly active role in their care [5]. Our findings highlight that though it may be hard to anticipate what will be concerning to patients, it is important for healthcare providers to give patients and families the opportunity to bring these issues up.

When conversations about ethically challenging situations were considered comfortable and helpful, respondents primarily pointed to the content and quality of communication as facilitating factors (Table 6). Wanting more information was the most frequent response in each of the open-ended questions. For patients and family members facing ethically challenging situations where they are not sure what is the “right” thing to do, clear information regarding the disease process, prognosis, treatment options, and risks and benefits is paramount in helping decision-making [18, 19]. Surveys, including our own, show that most patients and families wish to receive as much information as possible, perhaps to cope with uncertainty [20–22].

Notably, a subset of respondents did not find the discussion regarding their ethical concerns helpful and/or comfortable. Respondents’ suggestions for improving the comfort and helpfulness of discussions (Tables 5 and 7) correspond well to recommendations in the literature for clinicians when they are called upon to have difficult conversations, such as giving bad news and initiating discussions about palliative or end-of-life care [23, 24]. The evidence from our survey suggests that clinicians ought to be prepared to have discussions about ethically challenging concerns that arise throughout the life cycle of patients, not just at the end-of-life. Some skills that are essential to navigating difficult conversations include asking open-ended questions (exploration) to gauge a patient’s or family member’s knowledge before giving information (asking before telling), listening without interrupting, and addressing emotions with empathy (feelings before facts) [23–25].

Empathy was one prominent healthcare provider quality that respondents mentioned time and time again to make discussions regarding ethical concerns more comfortable and helpful (Tables 5 and 7). Patients and their families often enter clinical environments at extraordinarily stressful and traumatic moments in their lives, facing medical uncertainty and difficult decision-making. Empathy is considered one of the most powerful ways of providing support to patients and families by reducing their feelings of isolation and validating their thoughts and feelings [24].

When accounting for what would have made conversations about ethical concerns more comfortable and helpful, a large number of respondents mentioned contextual factors apart from communication quality and healthcare provider characteristics (Tables 5 and 7). Many pointed to providing sufficient resources or referrals—these findings recognize and affirm the importance of the larger healthcare team. Some respondents felt emotionally unprepared or did not feel like they had the health literacy to navigate these difficult conversations. Others felt like having a previously established relationship would have been made these discussions more comfortable and helpful. There was also a significant number of respondents who wanted the involvement of their family in these ethically challenging discussions, because they rely on familial relationships to help them feel supported or make decisions. This finding supports a growing recognition that appropriately engaging the family, if a patient so desires, can augment respect for a patient’s autonomy [26].

Our study has several limitations, as described in Cho et al. 2020, including variable response rates; one geographic region; selected inpatient areas; exclusion of patients who did not speak English or were in distress, eating, sleeping, in isolation or with a provider; a cross sectional survey; and the possibility of recall bias [4]. In addition, since we asked participants about the most memorable ethical concern they faced, it may be unsurprising that such a high percentage of them spoke to a healthcare provider about these concerns. In instances patients and families recall less well, which may have been less emotionally challenging or impactful, the percentage who spoke to a healthcare provider might be lower.

## Conclusion

This study offers insights into the perspectives of patients and family members regarding communication with healthcare providers when they face ethically challenging circumstances during care. Our data highlight that when patients and families are uncertain about how to respond to their own or a family member’s illness and about the right decision to make, they find it helpful to discuss their concerns and seek advice from healthcare providers. Our study underscores the need for providers to be prepared to talk to patients and families when they are concerned or uncertain and the ways to help them feel comfortable to have such difficult discussions.

## Supplementary Information

**Additional file 1. **Qualitative Code Descriptions.

**Additional file 2. **Associations Between Attitudinal Factors and Respondent Sociodemographic Variables.

**Additional file 3. **Associations Between Speaking to a Healthcare Provider and Healthcare Provider/Respondent Variables.

**Additional file 4. **Associations Between Comfortable Discussion and Healthcare Provider/Respondent Variables.

## Data Availability

The datasets used and/or analyzed during the current study are available from the corresponding author on reasonable request.
