# Supplementary material for: Communication of patients’ and family members’ ethical concerns to their healthcare providers
Source: BMC Med Ethics. 2023 Jul 29;24:56. doi: 10.1186/s12910-023-00932-x (PMC10385941; doi:10.1186/s12910-023-00932-x)
Supplement: Supplementary file 1 — Additional file 1. Qualitative Code Descriptions. [file 12910_2023_932_MOESM1_ESM.docx]

**Additional file 1. Qualitative Code Descriptions**

| **What, if anything, would have made you MORE comfortable talking with your healthcare providers about this situation?** | | |
| --- | --- | --- |
| **Codes** | **Sub codes** | **Examples** |
| Contextual factors  Refers to the context of the situation (i.e. not something necessarily inherent in the exchange) | A= Having adequate time/timing  B= Providing sufficient resources or referrals  C= Being prepared or ready to have the discussion  D= Having a previously established relationship with the healthcare provider  E= Having privacy  F= Having family present or involved  G=Other | A= “Having more time to think about the situation”; “The doctors having more time to spend with each patient”  B= “If there were more readily available resources for our providers to be able to assist”  C= “being more prepared, knowing more about advanced directives”  D= “having a previous relationship or knowing them better”  E= “Being able to ask questions in private and not involve family members who had no business being involved”  F= “I didn't have my daughter there. I wish my daughter had been there for better info about the visit”  G= “coming to a conclusion rather than more tasks and events to occur in order to complete advanced directives. Healthcare providers are the start, but rarely able to help the patient prepare a final advance directive that covers all their needs” |
| Healthcare provider qualities | A= More empathetic  B=More open-minded  C= More knowledgeable and experienced  D= More honest and transparent  E= More trustworthy  F= Other | A= “feeling more like they cared about me as an individual and not just ‘another patient’”  B= “If she had taken our advocacy more seriously and less defensively”  C= “If they know what the situation was”  D= “If they had been comfortable enough to be honest about what they know, what they could or could not do, about what the treatments + probable outcomes would really involve…”  E= “introduced themselves, made her comfortable, ask her what they could do for her, trust building”  F= “I never felt they were confident in their answers” |
| Content and/or quality of communication | A= Providing more or different information  B= More listening (attentive communication)  C= Better or clearer communication  D= More proactive communication  E=Other | A= “If the healthcare providers provide more information”  B= “If they would listen to me more and not think I just want drugs for pain”  C= “options laid out more clearly. Better communication”  D= “They could have told me sooner…”  E= “Talking in my language to explain the problem very well” |

| **Why did you find this discussion(s) with the healthcare provider(s) helpful?** | | |
| --- | --- | --- |
| **Codes** | **Sub codes** | **Examples** |
| Content and/or quality of communication | A= Provided helpful information (answers to questions, explanations, options, information, and/or understanding)  B= Communicated well or clearly  C= Listened (attentive communication)  D= Other | A= “Because it informed me of my options and the benefits of advanced medical equipment and treatments”  B= “They do well with communicating with people who don't have a medical background”  C= “By hearing what I had to say”  D= “Because at least people are talking about it” |
| Healthcare provider qualities | A= Knowledgeable or experienced  B= Empathetic  C= Honest or transparent  D= Patient  E= Trustworthy  F= Put respondent at ease  G= Open-minded  H= Other | A= “He had a lot of experience dealing with the things I was going through”  B= “Because they understood the situation and showed empathy and compassion”  C= “always been truthful. Didn't hold anything back”  D= “He took time for me to look at my notes and I got to ask every single question”  E= “Because I trust them when [genuine]”  F= “Put patient at ease with her sons & husband situation.”  G= “Open to ideas from family”  H= “he was encouraging” |
| Helpful action | A= Assisted with decision-making  B= Facilitated access to helpful resources  C= Intervened effectively  D= Other | A= “They helped us to decide a plan of care that honored my mom as best as possible”  B= “they helped me find resources to help with my dad”  C= “modifications were made to my meds to match what it should be”  D= “Because all of my issues and concerns were addressed” |
| Time and availability of the healthcare provider | A= Time and availability | A= “…spent enough time with me” |

| **Why didn’t you find this discussion(s) with the healthcare provider(s) helpful?** | | |
| --- | --- | --- |
| **Codes** | **Sub codes** | **Examples** |
| Content and/or quality of communication | A= Did not provide helpful information  B= Did not listen (inattentive communication)  C= Communicated unclearly  D= Did not actually communicate  E= Other | A= “not enough information”  B= “He did not listen to pleas for alternative solutions”  C= “Options were not clear”  D= “I never did talk directly w. the doctor…”  E= “We were roadblocked told nurses hands were tied. Paged doctors for hours w/no response. When doctors arrived they blamed each other for no treatment” |
| Healthcare provider qualities | A= Single-minded or dismissive  B= Unempathetic  C= Not knowledgeable or experienced  D= Untrustworthy  E= Hurrying  F= Other | A= “She was one sided, had a one track mind”  B= “He was very cold and matter of fact in his communication. It was just a routine for him”  C= “She didn't know anything about psychology or medicine”  D= “Did not trust him…”  E= “…a ‘gotta go home see you tomorrow’ feeling”  F= “They acted as if they were afraid of him because he was hiv +” |
| Contextual factors | A= Not reaching a solution  B= Lack of resources  C= Lack of privacy  D= Other | A= “Nothing was resolved, no diagnosis”  B= “…they can't help with financial issues”  C= “…breached my privacy”  D= “…he prescribed medication that still caused side effects & did not work” |

| **What, if anything, would have made this discussion MORE helpful?** | | |
| --- | --- | --- |
| **Codes** | **Sub codes** | **Example** |
| Content and/or quality of communication | A= Providing more or different information  B= Better or clearer communication  C= More listening  D= Other | A= “A little more information on condition of mother”  B= “…less 'medical' terminology; less conflicting advice”  C= “for the doctor to listen to us”  D= “something that could have been done to talk with patient” |
| Healthcare provider qualities | A= More empathetic  B= More knowledgeable and experienced  C= More open-minded of concerns  D= More honest and transparent  E= More patient  F= Other | A= “If he had cared”  B= “…Being connected with [knowledgeable] people concerning these issues”  C= “Weighing my concerns with the same objectivity as I considered theirs”  D= “If healthcare practitioners would have been more direct about his prognosis”  E= “If healthcare provider had more patience”  F= “A different physician - maybe younger and of color. I'm being honest” |
| Contextual factors | A= Having adequate time/timing  B= Providing sufficient resources or referrals  C= Being prepared or ready to have the discussion  D= Having a previously established relationship with the healthcare provider  E= Having privacy  F= Having family present or involved  G= Other | A= “PCP- don't know how much time- always feel rushed”  B= “…perhaps they could refer me to a "gateway" person... who could then help identify the right person... to go to or at least a specific next step to find the right person. Just handing out a list of psychologists/psychiatrists doesn't help”  C= “Previous research and a [foundational] knowledge of the advance directive”  D= “Having a longer doctor patient relationship”  E= “more privacy…”  F= “having my [mom’s] input”  G= “That the conversation with the healthcare provider would be the final conversation about the talk of advanced directive…” |
