# Supplementary material for: Communication of patients’ and family members’ ethical concerns to their healthcare providers
Source: BMC Med Ethics. 2023 Jul 29;24:56. doi: 10.1186/s12910-023-00932-x (PMC10385941; doi:10.1186/s12910-023-00932-x)
Supplement: Supplementary file 2 — Additional file 2. Associations Between Attitudinal Factors and Respondent Sociodemographic Variables. [file 12910_2023_932_MOESM2_ESM.docx]

**Additional file 2. Associations Between Attitudinal Factors and Respondent Sociodemographic Variables**

| Attitudinal Factor | Respondent Sociodemographic Variable | Chi-square p-value | Category with Highest % Agreed | Category with Highest % Disagreed |
| --- | --- | --- | --- | --- |
| Unsure whether situation important | Race/ethnicity | 0.0001 | Multiple (36.8%)  Black (33.6%) | White (88.7%) |
| HCP seemed very busy | Age | 0.0047 | 18-44  (70.9%) | 65+ (50.9%) |
| Wanted advice from HCP | Marital status | 0.0090 | Married/living with partner (80.7%) | Divorced/separated (43.5%) |
| HCP seemed kind | Gender identity | 0.0161 | Male (94.0%) | Female (14.1%) |
| HCP seemed kind | Health insurance | 0.0200 | Employer-sponsored (95.2%) | No insurance (30.0%) |
| HCP seemed trustworthy | Household income | 0.0246 | <$25,000 (88.5%) | $25,000-49,999 (29.6%) |
| HCP seemed trustworthy | Employment status | 0.0281 | Employed (89.1%) | Unemployed (19.6%) |
| HCP was easy to understand | Religious preference | 0.0355 | Other (94.9%) | No religious preference (25.4%) |
| HCP seemed very busy | Household income | 0.0360 | $100,000+ (72.2%) | $25,000-$49,999 (49.1%) |
| Felt powerless to voice opinion | Health insurance | 0.0375 | Military/veteran (50.0%) | Employer-sponsored (81.9%) |

*160 univariable analyses were done, but only those with p-values that are less than 0.05 are included in this table. 0.05 was chosen rather arbitrarily, simply to cut down on the length of the table. After adjusting for multiplicity by using a p-value threshold of 0.0003 (=0.05/(10x16)), the evidence from associations with p-values greater than this value should be considered as weak. The adjusted p-value threshold of 0.0003 should be used as a very rough guideline for the strength of the evidence of a possible association.
