# Supplementary material for: Communication of patients’ and family members’ ethical concerns to their healthcare providers
Source: BMC Med Ethics. 2023 Jul 29;24:56. doi: 10.1186/s12910-023-00932-x (PMC10385941; doi:10.1186/s12910-023-00932-x)
Supplement: Supplementary file 3 — Additional file 3. Associations Between Speaking to a Healthcare Provider and Healthcare Provider/Respondent Variables. [file 12910_2023_932_MOESM3_ESM.docx]

**Additional file 3. Associations Between Speaking to a Healthcare Provider and Healthcare Provider/Respondent Variables**

| Explanatory variable | p-value | AUC | AUC 95% CI lower limit | AUC 95% CI upper limit |
| --- | --- | --- | --- | --- |
| Number of positive respondent attitudinal factors | 0.0009 | 0.62 | 0.55 | 0.69 |
| Gender identity | 0.15 | 0.54 | 0.48 | 0.60 |
| Age | 0.17 | 0.56 | 0.50 | 0.62 |
| Birthplace | 0.24 | 0.52 | 0.48 | 0.56 |
| Employment status | 0.27 | 0.53 | 0.48 | 0.59 |
| Number of negative respondent attitudinal factors | 0.35 | 0.54 | 0.47 | 0.61 |
| Health insurance (amount) | 0.45 | 0.53 | 0.49 | 0.57 |
| Marital status | 0.47 | 0.56 | 0.50 | 0.62 |
| Household income | 0.59 | 0.55 | 0.48 | 0.61 |
| Education | 0.91 | 0.51 | 0.45 | 0.57 |
| Number of positive healthcare provider attitudinal factors | 0.92 | 0.52 | 0.45 | 0.57 |
| Religious preference | 0.95 | 0.53 | 0.47 | 0.59 |
| Health insurance (type) | 0.95 | 0.52 | 0.46 | 0.58 |
| Number of negative healthcare provider attitudinal factors | 0.98 | 0.52 | 0.45 | 0.60 |
| Race/Ethnicity | 0.98 | 0.54 | 0.48 | 0.60 |
